# Lactic Acid Bacteria Isolates and the Microbiome of Cincalok, Tempoyak, and Mandai: A Traditional Fermented Food from Kalimantan Island, Indonesia

**DOI:** 10.1155/2024/6589766

**Published:** 2024-04-05

**Authors:** Retno Murwani, Refa Anggraeni, Gregorius Nico Adi Setiawan, Putri Dyah Astari, Ni Kadek Dita Cahyani, Mada Triandala Sibero, Ambariyanto Ambariyanto

**Affiliations:** ^1^Natural Product Laboratory-UPT-Laboratorium Terpadu, Universitas Diponegoro, Semarang, Central Java, Indonesia; ^2^Laboratory of Physiology and Biochemistry, Faculty of Animal and Agricultural Sciences, Universitas Diponegoro, Semarang, Central Java, Indonesia; ^3^Biology Department, Faculty of Science and Mathematics, Universitas Diponegoro, Semarang, Central Java, Indonesia; ^4^Marine Biodiversity Research Project, UPT-Laboratorium Terpadu, Universitas Diponegoro, Semarang, Indonesia

## Abstract

Indonesia has abundant traditional fermented food with various lactic acid bacteria (LAB), which can be developed into probiotics for pharmaceutical and functional food and feed products. This research is aimed at (1) obtaining and identifying LAB isolates and (2) studying the microbiome (bacterial diversity and abundance) of spontaneously-fermented traditional foods of Kalimantan Island, Cincalok, Tempoyak, and Mandai. To obtain LAB isolates, food samples were serially diluted and inoculated on MRS agar that contained 1% CaCO_3_ (MRSA). Isolates forming clear zones were purified and identified by DNA barcoding. The microbiome was studied using genomic-sequencing techniques and analysed for taxonomic composition. Seven pure isolates were obtained from Cincalok, two Tempoyak, and one Mandai. DNA barcoding revealed that the Cincalok seven isolates were *Staphylococcus carnosus* (strain HSP-S16), *Tetragenococcus halophilus* (FSB201), *Corynebacterium phoceense*, *Vagococcus vulneris* (SS1995), *Enterococcus faecalis* (S11-6), *Pisciglobus halotolerans* (C01), and *Priestia filamentosa* (P3.1); two from Tempoyak, *Levilactobacillus brevis* (E1D3BL1) and *Lactiplantibacillus plantarum* (UMCC-2996); and one from Mandai, *Staphylococcus cohnii* (XAAS.x13; non-LAB). The *T. halophilus*, *E. faecalis*, *P. halotolerans*, *L. brevis*, and *L. plantarum* belong to LAB. The *P. halotolerans* from Cincalok and non-LAB in these three fermented foods were the first documented report. The microbiome revealed the dominance of *Firmicutes* phyla in the fermented foods, with 93% in Cincalok, 89.94% in Tempoyak, and 60.32% in Mandai. On the genus level, Cincalok was dominated by *Tetragenococcus* 40.33%, *Anaerococcus* 23.29%, *Vagococcus* 9.27%, and *Lactobacillus* 6.84%. Meanwhile, Tempoyak was dominated only by *Lactobacillus* 89.94%. Mandai were dominated by *Lactobacillus* 31.97%, *Proteus* 17.14%, *Aerococcus* 16.85%, *Mangrovibacter* 15.15%, and *Vagococcus* 6.2%. However, Mandai's microbiome LAB was not culturable/isolated on MRSA. The plausibility is that those unculturable LAB require coculturing with other bacteria and additional media components to grow on MRSA. This study is the first report regarding the microbiome of Cincalok, Tempoyak, and Mandai, along with their culturable LAB isolates.

## 1. Introduction

Lactic acid bacteria (LAB) have been widely studied as potential candidates for probiotic agents [1–3]. Some genera, such as *Enterococcus*, *Lactobacillus*, *Pediococcus*, and *Lactococcus*, exhibit probiotic properties that can potentially treat several bacterial infections and develop into functional food and feed products. These genera could be isolated from the intestinal tract, rumen, fermented beverages, and foods [4–6]. Fermented foods are a rich source of LAB and probiotics [7]. Probiotic bacteria are characterized by their ability to tolerate gastric and intestinal juice, inhibit several pathogenic bacteria (competitive exclusion, antiadherence, and anti-infectivity), capable of adhering to intestinal cells, and have no DNase or hemolytic activity as an indicator of their safety [3]. As a multicultural country, Indonesia has plenty of traditional fermented foods that preserve the abundance of LAB. Three of them are Cincalok, Tempoyak, and Mandai.

Cincalok is a fermented traditional food from West Kalimantan, Indonesia. In South Kalimantan, it is known as Ronto, in Malaysia as Cincaluk, and in the Philippines as Bagoong Alamang. Cincalok is made of small shrimp (*rebon*) as the main ingredient, fermented spontaneously using salt only. However, a study used additional sugar and several spices [8]. Tempoyak, made of durian (*Durio* sp.), is a typical traditional functional food made and consumed by local communities in several locations on the Sumatra and Kalimantan islands [9]. Another fermented food is Mandai, a chewy snack from South Kalimantan, which is made of fermented inner skin flesh of jackfruit *Artocarpus integer* [10]. Previous studies have found LAB from Cincalok, i.e., *Weissella* sp. using DNA barcoding but no isolation [11], *Staphylococcus* (*S*) *piscifermentans* (isolation) [12], *Pediococcus* (*P*) *halophilus* and *P. dextrinicus* [13], *L. rhamnosus*, *S. piscifermentans*, and *S. saprophyticus* [14]. Tempoyak had been found to harbor the following LAB: *E. gallinarum* and *E. faecalis* [15]; *Lb. plantarum*, *P. acidilacti*, and *Weissella paramesenteroides* [16]; *Fructobacillus* (*F*) *durionis*, *L. plantarum*, *Leu. mesenteroides*, *L. brevis*, *L. collinoides*, *L. casei*, and *L. fructivoran*s (DNA barcoding, no isolates) [17]; and *L. plantarum*, *L. acidophilus*, *L. brevis*, and *L. buchneri* [18]. Meanwhile, Mandai harbors *L. plantarum* [19], *L. plantarum*, *L. monocytogenes*, and one non-LAB isolate *S. Typhimurium* [20]. However, compared to other traditional Indonesian fermented foods, the LAB in Cincalok, Tempoyak, and Mandai are less reported.

LAB has a great deal of application possibilities in product development. Probiotics as food additives are still greatly needed to maintain sustainability and good health. LAB has been noted to have immunostimulant effects in animals and humans. They are the most widely used microorganisms as a potential source of probiotics. Their uses include pharmaceutical preparations as a functional food to improve public health [21, 22]. Some LAB were reported to protect their host from pathogens and parasites, decrease blood sugar levels, reduce cholesterol assimilation, and prevent various diseases [3, 23]. In addition, it has nondisease properties and interacts with the gastrointestinal microbiota and the immune system [21–23]. Therefore, studying the LAB diversity from traditional Indonesian fermented foods, i.e., Cincalok, Tempoyak, and Mandai, is valuable. Moreover, the microbiome (microbial diversity and abundance) in Cincalok, Tempoyak, and Mandai by genomic sequencing has never been studied.

## 2. Materials and Methods

The research was conducted at the Natural Product Laboratory-UPT-Laboratorium Terpadu, Universitas Diponegoro, Semarang. The study is divided into isolation, isolate characterization, and identification by molecular approach. Furthermore, genomic sequencing was carried out to study the microbiome (bacterial diversity and abundance) of Cincalok, Tempoyak, and Mandai (whole food).

### 2.1. Sample Collection

Traditionally made Cincalok (CC) were purchased from a traditional market, Pangkalan Bun, and a Food shop, Banjarmasin. Tempoyak (T) and Mandai (M) were purchased from Banjarmasin Tengah, Banjarmasin City, Kalimantan. The products were spontaneously fermented using salt only and stored in closed food containers.

### 2.2. Isolation and Screening of Lactic Acid Bacteria (LAB) Candidates

De Man-Rogosa-Sharpe (HiMedia, India) agar that contains 1% CaCO_3_ (MRSA) was prepared to isolate LAB from the food samples. Bacterial isolation was carried out using the serial dilution method [24–27]. One gram of each food sample was transferred into 9 mL of physiological saline solution (0.9% NaCl) to obtain the first dilution (10^−1^). The dilution continued to reach 10^−7^. Afterward, 50 *μ*L of each dilution was transferred onto MRSA, spread using a glass spreader, and incubated aerobically and anaerobically at 37°C for 1‐2 × 24 h until colony growth was observed. The anaerobic condition was obtained by incubating the plate in a plastic vacuum bag (KRIS). The sample dilution method for isolating LAB candidates was done in triplicates. The colonies were purified by observation of a clear zone formation around the colonies. A clear zone formation indicates that the colonies produced acids that react with the CaCO_3_ in the medium. All overlapping and finely separated colonies from each dilution were purified using a sterile inoculating loop and streaked using the standard four quadrants on a new MRSA. After repeated purification, all pure isolates that grow on MRSA and form a clear zone were reobserved for their morphology (form, color, and texture) and ability to grow aerobic and anaerobically (Table 1) to screen LAB candidates. The pure isolates were streaked to a new MRSA by continuous method for identification of LAB using the molecular method (DNA barcoding).

### 2.3. Identification of LAB Isolates Using DNA Barcoding

The DNA of the pure isolates was extracted using Quick DNA Fungal/Bacterial ZymoBIOMICS™ MiniPrep Kit (Zymo Research D4300, USA) and additional incubation with proteinase K (Zymo Research D3001-2-20, USA). The PCR mixture consisted of 12.5 *μ*L GoTaq® Green Master Mix (Promega, USA), 1 *μ*L DNA template, 1 *μ*L primer forward, 1 *μ*L primer reverse, then ddH_2_O (Zymo Research, USA) until a total of the final mixture reached 25 *μ*L. Primers 27F (5′-AGA GTT TGA TCM TGG CTC AG-′) and 1492R (5′-GGT TAC CTT GTT ACG ACT T-3′) were applied for bacteria [26]. Amplification by PCR using (2x) My Taq HS Red Mix (Bioline, BIO-25048) was performed with the following conditions: initial denaturation at 95°C for 3 min, denaturation at 95°C for 15 sec, annealing at 52°C for 30 sec, extension at 72°C for 45 sec for 35 cycles, and final extension at 72°C for 3 min. Gel electrophoresis in 1% TBE agarose with a 1 kb DNA ladder was carried out to check the PCR product. The PCR product was sequenced by bidirectional sequencing using BigDye® Terminator v3.1 Cycle Sequencing Kit on ABI PRISM 3730xl Genetic Analyzer (Applied Biosystems, USA) at 1st BASE Laboratories, Apical Scientific Sdn. Bhd., Selangor, Malaysia. Then, the sequence was used to determine the species by comparing it to GenBank data using the Basic Local Alignment Search Tool (BLAST) in NCBI. The phylogenetic tree was reconstructed using the MEGA X software package [27].

### 2.4. Microbiome of Cincalok, Tempoyak, and Mandai by Genomic Sequencing

DNA extraction of Cincalok, Tempoyak, and Mandai and subsequent PCR and sequencing were done in a sequencing facility using next-generation sequencing following the amplicon-based approach. Bacterial 16S rRNA gene regions (V4) were amplified using specific primers 515F-806R with the barcode. All PCR reactions were carried out with Phusion® High-Fidelity PCR Master Mix (New England Biolabs, USA). The same volume of 1x loading buffer (containing SYB green) was mixed with PCR products, and electrophoresis was carried out on 2% agarose gel for detection. Samples were sequenced using the Illumina platform. Amplicon was sequenced on the Illumina paired-end platform to generate 250 bp paired-end raw reads (raw PE) and then merged and pretreated to obtain clean tags. The microbial community composition in each sample was studied using Operational Taxonomic Units (OTUs) obtained through clustering with 97% identity on the effective tags of all samples. Subsequently, these OTUs were identified using the SILVA SSU nonredundant database (https://github.com/qiime2/q2-feature-classifier). The taxonomic bar plots were constructed by selecting the top 10 taxa of each sample or group at each taxonomic rank (phylum and genus) to form the distribution histogram of the relative abundance of taxa.

## 3. Results

### 3.1. Isolation and Screening of Lactic Acid Bacteria (LAB) Candidates, their Morphology, and Aerobic and Anaerobic Growth

**Figure 1:**
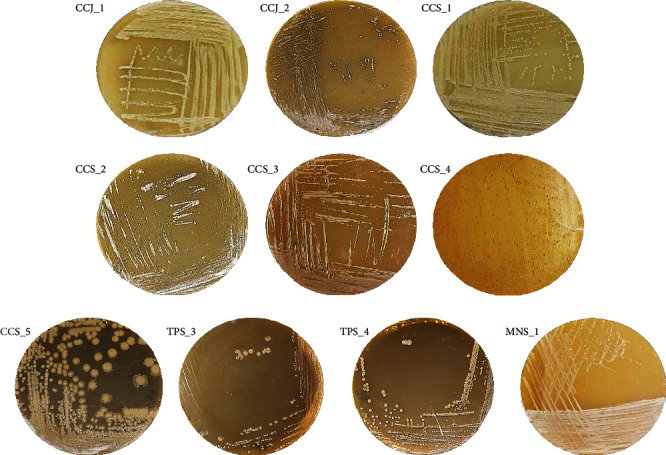
Pure bacterial isolates from Cincalok, Tempoyak, and Mandai on MRS agar.

Isolation of LAB from fermented foods Cincalok, Tempoyak, and Mandai produced ten pure isolates (Figure 1) coded with abbreviations according to the food source. Seven isolates, CCJ_1, CCJ_2, CCS_1, CCS_2, CCS_3, CCS_4, and CCS_5, were obtained from Cincalok; two isolates, TPS_3 and TPS_4, were from Tempoyak; and one isolate, MNS_1, from Mandai.

Cell and colony macroscopic morphology show diverse colors, shapes, elevations, and edges (Table 1). CCJ_1, TPS_3, and MNS_1 had the same morphological characteristics of a white colony color, circular colony shape, convex elevation of colonies, and entire or perfectly round edges. However, as they came from different food samples, they were considered different bacterial types and further identified by DNA barcoding. CCJ_2 isolate has morphological characteristics of creamy white colony color, perfectly round (circular) colony shape, the convex elevation of colonies, and entire or perfectly round edges. CCS_1 isolate has morphological characteristics of white colony color, perfectly round (circular) colony shape, convex elevation (protruding center), and entire or perfectly round edges. CCS_2 isolate has morphological characteristics of white colony color, circular colony shape, flat elevation of colonies, and entire or perfectly round edges. CCS_3 isolate has a white colony color, circular colony shape, prominent elevation of colonies, and entire or perfectly round edges. CCS_4 isolate has a yellow colony color, perfectly round (circular) colony shape, prominent elevation of colonies, and entire or perfectly round edges. TPS_4 isolate has a white colony color, circular colony shape, convex elevation of colonies, and entire or perfectly round edges. CCS_5 appeared to secrete secondary metabolites into the media so that the media changed color. At a young age, CCS_5 is gray; in maturity, it is dark blue; when aged, it is black. The growth of isolates on MRSA media under aerobic and anaerobic conditions shows similarities except CCJ_2 (Table 1).

### 3.2. Bacterial Isolate Identification by DNA Barcoding

**Figure 2:**
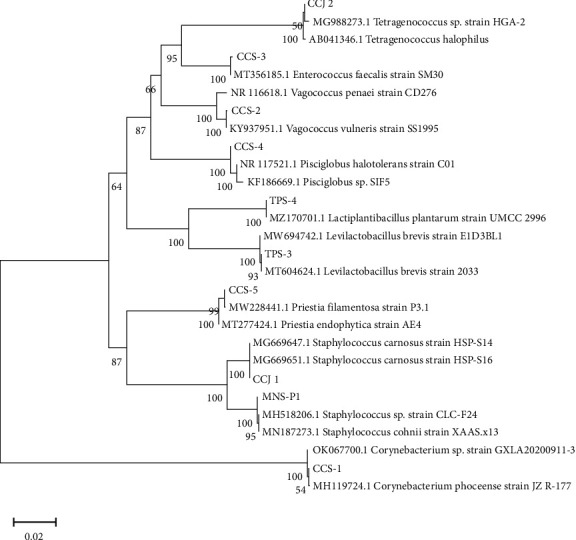
The phylogenetic tree of the ten identified isolates from Cincalok (CC), Tempoyak (T), and Mandai (M).

The molecular identification results of the isolates are shown in Table 2, and the sequencing results with the phylogenetic tree are shown in Figure 2.

The DNA barcoding approach successfully discovered the existence of some LAB from Cincalok and Tempoyak, namely, *Tetragenococcus* (*T*) *halophilus* (CCJ_2), *E. faecalis* (CCS_3), *P. halotolerans* (CCS_4), *L. brevis* (TPS_3), and *L. plantarum* (TPS_4), and together with five non-LAB, namely, *S. carnosus* (CCJ_1), *Corynebacterium phoceense* (CCS_1), *Vagococcus vulneris* (CCS_2), *Bacillus filamentosus* (CCS_5), and *S. cohnii* (MNS_1). It is noted that Cincalok had more diverse bacterial genera than Tempoyak.

### 3.3. Microbiome of Cincalok, Tempoyak, and Mandai by Genomic Sequencing

**Figure 3:**
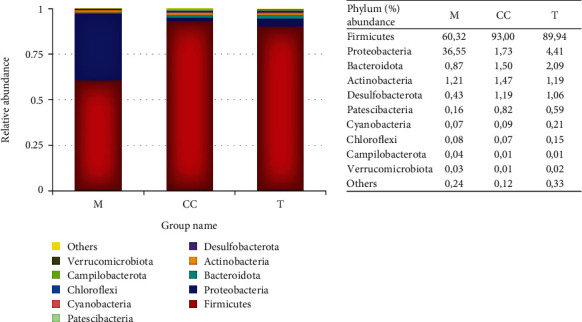
Microbiome (bacterial diversity and abundance) in Cincalok (CC), Tempoyak (T), and Mandai (M) at the phylum level.

**Figure 4:**
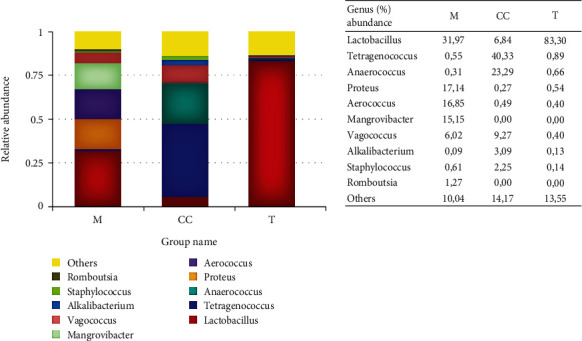
Microbiome (bacterial diversity and abundance) in Cincalok (CC), Tempoyak (T), and Mandai (M) at the genus level.

The genomic sequencing resulted in 798,764 reads and 1,347 OTUs of bacterial taxa. *Firmicutes* dominated the sample on the phylum level, with 93% in Cincalok, 89.94% in Tempoyak, and 60.32% in abundance in Mandai (Figure 3). Mandai was also dominated by 36.55% of *Proteobacteria*. On the genus level, Mandai were dominated by *Lactobacillus* 31.97%, *Proteus* 17.14%, *Aerococcus* 16.85%, *Mangrovibacter* 15.15%, and *Vagococcus* 6.2%. Cincalok was dominated by *Tetragenococcus* 40.33%, *Anaerococcus* 23.29%, *Vagococcus* 9.27%, and *Lactobacillus* 6.84%. Meanwhile, Tempoyak was dominated by *Lactobacillus* 89.94% (Figure 4).

## 4. Discussion

Fermentation technology is one of humans' most ancient food-preserving techniques. It has been renowned that fermented foods are less perishable than raw materials, their nutritional value is enhanced, and the safety of the foods is improved due to the inhibition of pathogenic bacteria by the low pH from the presence of bacterial organic acids, high salt concentration, and production of antimicrobial compounds. In addition, organic acids and natural antimicrobial compounds are produced by LAB [28]. Moreover, the LAB is also known to be responsible for food flavor and texture diversity due to many factors. The variety comes from the dynamic during the fermentation process and the diversity of raw food material, whether the same or different.

Indonesia has many traditional foods that use fermentation in manufacturing [29]. Cincalok, Tempoyak, and Mandai are three famous traditional fermented foods found in several regions of Indonesia, including Kalimantan Island [8, 10, 30]. Cincalok, Tempoyak, and Mandai are typical traditional fermented foods that depend on spontaneous fermentation from the indigenous microbes from the raw material, including the LAB. These microbes degrade the macronutrient into smaller substances that give unique flavor and aroma to the product [31]. The current work isolated the LAB using MRS agar with 1% CaCO_3_ (MRSA). Therefore, their ability to secrete acid and neutralize CaCO_3_ is a selective method to obtain LAB [32]. Several studies added additional substances to create more diverse LAB using MRS and MRS with 2% fructose and obtained some species from *Enterococcus*, *Lactobacillus*, *Lactococcus*, *Leuconostoc*, and *Weissella* [33]. A previous study successfully isolated some LAB and one non-LAB from a cow's rumen using MRSA [27]. Another study isolated genera *Bacillus*, *Brevibacillus*, and *Streptococcus* from finger millet using MRSA [34]. Seven pure bacterial isolates were successfully obtained from Cincalok, two from Tempoyak, and one from Mandai (Table 1). Interestingly, Cincalok had three LAB genera, while Tempoyak only had two. A similar study reported that fermented shrimp had a higher microbial diversity than fermented plants [35]. The type and quality of raw material, manufacturing process, and climate significantly impact the LAB diversity in a fermented product [36–38].

The ten identified pure isolates of CCJ_1 as *S. carnosus*, CCJ_2 as *T. halophiles*, CCS_1 as *Corynebacterium phoceense*, CCS_2 as *V. vulneris*, CCS_3 as *E. faecalis*, CCS_4 as *Piscoglobus halotolerans*, CCS_5 as *P. filamentosus*, TPS_3 as *L. brevis*, TPS_4 as *L. plantarum*, and MNS_1 as *S. cohnii* showed similar colony morphology with the respective identified species found in a dry sauce [38], condensed sugar [39], human urine [40], human foot wounds [41], in survey samples [42], fish sauce [43], the rhizosphere [44], plant-based Thai fermented food [45], various fermented foods [46], and human skin [47], respectively. From the ten identified isolates, *T. halophilus*, *E. faecalis*, and *P. halotolerans* from Cincalok and *L. brevis* and *L. plantarum* from Tempoyak belong to LAB. The LAB can grow under aerobic and anaerobic conditions at a predetermined time. The ability of LAB to grow in anaerobic and aerobic conditions has been known [48]. LAB generally live under anaerobic conditions or do not require oxygen but can grow under aerobic conditions (facultative anaerobic) to produce pyruvic acid into acetyl phosphate and acetate and convert oxygen into peroxide [49]. Some LAB that grow in aerobic conditions undergo respiration metabolism mediated by a heme-dependent cytochrome oxidase [50]. Aerobic respiration is more significant to increase biomass yield but does not increase growth rate [51]. However, respiration metabolism increases long-term survival, which is essential for LAB application [49, 52].

The five LAB isolates belong to common lactic acid bacteria in fermented foods [36, 37, 43, 53–56]. A previous Tempoyak study found *E. gallinarum*, *L. plantarum*, *P. acidilactici*, *W. paramesenteroides* [16, 30], and *L. brevis* [57, 58]. *E. faecalis* was also found in Iranian fermented milk kashk [6], while *L. brevis* was in Thailand fermented food [47], *L. plantarum* in buffalo milk [59], growol [60], kashk [6], and pickled bamboo shoots [61]. Interestingly, the present study is the first report of *P. halophilus* in Cincalok. A previous study found *T. halophilus* in thick sugar [39] while *P. halotolerans* in fish sauce [43].

Although *S. carnosus*, *V. vulneris*, *C. phoceense*, *P. filamentosus*, and *S. cohnii* are not LAB, the coexistence of these bacteria in fermented food is widely reported. Moreover, *S. carnosus* was verified as nonpathogenic and has been applied in meat fermentation [62] and sausage fermentation since 1950 [63]. Along with LAB, *S. carnosus* reduced unfavorable odor and improved a mutton jerky's physicochemical properties [64]. *Vagococcus vulneris* was found in human wounds [41], and as of today, this is the first species found in fermented food. *C. phoceense* was found to be a new pathogen in human urine [40, 65]. *P. filamentosus* was found in the desert of Cholistan, Pakistan [66], and sea sediment in Charao Island, Goa, India [67], and up to date, it has not been found in fermented food. *S. cohnii* was found in Semarang waters [68] and human skin [47], and it is an opportunistic pathogen [69]. The finding of pathogenic non-LAB could indicate the possible contamination in the fermented foods. Such non-LAB was widespread in many studies [70–73]. Moreover, the non-LAB also belongs to *Firmicutes* phyla, which cover a wide range of sources from soil and aquatic environments, the microbiome of human and animal guts, and some pathogens of humans, animals, and plants [74]. The genomic sequencing results also show the presence of non-LAB (Figure 4). One method to suppress pathogenic bacteria in spontaneously fermented foods was sterilizing the raw materials and using defined cultures [75].

The microbiome diversity revealed that the three fermented foods from Kalimantan showed similar dominance of the *Firmicutes* phyla. Cincalok has the highest abundance of *Firmicutes*, followed by Tempoyak and Mandai (Figure 3). *Firmicutes* play a significant role in the relationship between gut bacteria and human health [74]. Many of this phylum members (*Bacilli* class, order *Lactobacillales*, *Lactobacillaceae* family, and *Lactobacillus* genus) break down/ferment nondigestible nutrients such as dietary fiber and resistant starch in the gut. Small shrimp (fresh), as the raw material of Cincalok, has the highest protein content and lowest fiber [74]; inner skin flesh of jackfruit (dry powder), as the raw material of Mandai, has the highest cellulose (27.75%), with pectin (7.52 ± 0.12%), protein (6.27 ± 0.03%), and starch (4%) [76]. Durian fruit, the raw material of Tempoyak, has the highest carbohydrate (23.94-41.61%), with fructose as the predominant sugar, followed by glucose, fructose, and maltose [77, 78]. The specific durian variety contains more fructose than glucose [78]. Durian fruit also has low protein content. Interestingly, the highest *Firmicutes* phyla were found in Cincalok (Figures 3 and 4). However, at the genus level, *Tetragenococcus* was dominant, which aligns with the isolated species of *T. halophilus* from Cincalok. *Tetragenococcus* belong to facultative-aerobe LAB that can ferment melezitose, sucrose, maltotriose and xylose, galactose, glucose isomer, L-arabinose, D-manitol, maltose, and D-glycerol [79, 80]. The finding of *P. halotolerans* and *T. halophilus* indicated that the salt used in Cincalok provided conditions for salt-loving and tolerance LAB.

Meanwhile, on the genus level, *Lactobacillus* was dominant in Tempoyak and Mandai. The available sugars in durian as the raw material of Tempoyak appear to be favorable for LAB such as *L. brevis* and *L. plantarum*. The two species are widely found in many fermented foods with abundant sugars [6, 78]. Although *Lactobacillus* was dominant in Mandai, other genus, *Proteus*, *Aerococcus*, *Mangrovibacter*, and *Vagococcus*, were also present in approximately half of *Lactobacillus* abundance (Figure 4). *Proteus* was found in the digestive tract and fecal-contaminated water and was considered a contaminant and an indication of food processing hygiene [81, 82]. Meanwhile, *Aerococcus* also belongs to LAB that can produce acetic acid and hydrogen peroxide [83, 84]. Some *Aerococcus* sp. from dairy isolates can inhibit some strains of *Salmonella* in milk medium [84]. *Mangrovibacter* is usually found in wild rice roots that are associated with mangroves and ferment various sugars such as sucrose, raffinose, cellobiose, arabinose, sorbitol, glycerol, ribose, D-xylose, galactose, glucose, fructose, mannose, rhamnose, mannitol, methyl *α*-D-glucoside, N-acetylglucosamine, arbutin, cellobiose, maltose, melibiose, trehalose, gentiobiose, and L-fucose to produce acid [85]. The *Vagococcus* can produce organic and phosphoric acids from D-fructose [86]. Such microbiome abundance ratio may not provide suitable conditions for LAB found in Mandai to thrive alone on MRSA; therefore, it could not be obtained during repeated isolation on MRSA. A 16S rRNA gene sequencing (microbiome) study in kefir revealed that *Lactobacillus kefiranofaciens* was the dominant strain. However, unexpectedly, it was unable to grow in milk alone. Furthermore, when *L. kefiranofaciens* was cocultured with *Leuconostoc mesenteroides*, *L. kefiranofaciens* made amino acids available for *L. mesenteroides*, which in turn produced lactate available for *L. kefiranofaciens* [87]. Therefore, the LAB identified in Mandai by the genomic sequencing (microbiome study) may require coculturing with other bacteria and additional media components to be culturable on MRSA. It appears that the difference in the isolated LAB (culturable on MRSA) in this study was due to the raw material type (with different nutrient contents), the handling/processing of raw materials, the amount of added salt, and the length of fermentation, which affect the dynamic of bacterial diversity and their abundance and hence their balance within the food system (Cincalok, Tempoyak, and Mandai). Furthermore, MRS agar composition supports not only LAB but also other genera with acid-producing capability (aligned with the microbial diversity and abundance); therefore, non-LAB isolates were also obtained.

## 5. Conclusions

Ten bacterial strains were isolated from Cincalok, Tempoyak, and Mandai. Molecular identification showed that five were lactic acid bacteria. The LAB identified were *Tetragenococcus halophilus*, *Enterococcus faecalis*, *Pisciglobus halotolerans*, *Levilactobacillus brevis*, and *Lactiplantibacillus plantarum*. The phyla of *Firmicutes* dominated all three fermented foods, with 93% in Cincalok, 89.94% in Tempoyak, and 60.32% in Mandai. On the genus level, Cincalok was dominated by *Tetragenococcus* 40.33%, *Anaerococcus* 23.29%, *Vagococcus* 9.27%, and *Lactobacillus* 6.84%, aligned with the identified isolate *T. halophilus*. Meanwhile, Tempoyak was dominated only by *Lactobacillus* 89.94%, aligned with the two identified LAB isolates. Mandai were dominated by *Lactobacillus* 31.97%, *Proteus* 17.14%, *Aerococcus* 16.85%, *Mangrovibacter* 15.15%, and *Vagococcus* 6.2%. Further studies are required to develop modified methods for obtaining unculturable LAB from the three fermented foods. The probiotic property tests should be conducted on the five LAB isolates to develop into probiotic products.

## Figures and Tables

**Table 1:** The morphology and growth of the ten bacterial isolates from Cincalok, Tempoyak, and Mandai in Figure 1 at aerobic and anaerobic conditions.

Isolate code	Agar culture characteristic				Anaerobic (hours)					Aerobic (hours)				
	Colony color	Form	Elevation	Margin	24	48	72	96	120	24	48	72	96	120
CCJ_1	White	Circular	Convex	Entire	-	±	++	++	++	-	±	++	++	++
CCJ_2	Creamy white	Circular	Convex	Entire	-	-	-	-	±	-	-	-	-	±
CCS_1	White	Circular	Umbonate	Entire	-	±	±	±	++	-	±	±	±	++
CCS_2	White	Circular	Flat	Entire	-	±	±	±	++	-	±	±	±	++
CCS_3	White	Circular	Raised	Entire	-	±	±	±	++	-	±	±	±	++
CCS_4	Yellow	Circular	Raised	Entire	-	±	±	±	++	-	±	±	±	±
CCS_5	Gray, dark blue, and black	Irregular	Umbonate	Lobate	-	±	±	±	++	-	±	±	±	++
TPS_3	White	Circular	Convex	Entire	-	±	±	±	++	-	+	±	±	++
TPS_4	Yellow	Circular	Convex	Entire	-	±	±	±	++	-	±	±	±	++
MNS_1	White	Circular	Convex	Entire	-	±	±	±	++	-	±	+	+	++

Notes: -: negative growth; ±: weak growth; +: normal growth; ++: maximum growth.

**Table 2:** Molecular identification of pure isolates from Cincalok, Tempoyak, and Mandai grown in MRS agar.

Food source	Isolate code	Identified species	GenBank accession number of identified bacterial sequences
Cincalok	CCJ_1	*Staphylococcus carnosus*	OQ195272
	CCJ_2	*Tetragenococcus halophilus* (LAB)	OQ195275
	CCS_1	*Corynebacterium phoceense*	OQ195281
	CCS_2	*Vagococcus vulneris*	OQ195277
	CCS_3	*Enterococcus faecalis* (LAB)	OQ195276
	CCS_4	*Pisciglobus halotolerans* (LAB)	OQ195278
	CCS_5	*Priestia filamentosa*	OQ195274
	TPS_3	*Levilactobacillus brevis* (LAB)	OQ195279

Tempoyak	TPS_4	*Lactiplantibacillus plantarum* (LAB)	OQ195280

Mandai	MNS_1	*Staphylococcus cohnii*	OQ195273

## Data Availability

All the data is available in the manuscript.
